# Frequency and significance of parvovirus B19 infection in patients with rheumatoid arthritis

**DOI:** 10.1099/jgv.0.000621

**Published:** 2016-12-15

**Authors:** Milda Naciute, Diana Mieliauskaite, Rita Rugiene, Rita Nikitenkiene, Ligita Jancoriene, Mykolas Mauricas, Zaiga Nora-Krukle, Modra Murovska, Irute Girkontaite

**Affiliations:** ^1^​ Department of Immunology, State Research Institute Centre for Innovative Medicine, Santariskiu 5, Vilnius LT 08406, Lithuania; ^2^​ Centre of Rheumatology, Vilnius University, Vilnius, Lithuania; ^3^​ Department of Infectious, Chest Diseases, Dermatovenerology and Alergology and Hospital Santariskiu klinikos Centre of Infectious Diseases, Vilnius University, Vilnius, Lithuania; ^4^​ A. Kirchenstein Institute of Microbiology and Virology, Riga Stradins University, Riga, Latvia

**Keywords:** Parvovirus B19, rheumatoid arthritis, antibodies, IL-6

## Abstract

The present study aims to clarify the possible involvement of parvovirus B19 (B19V) infection in rheumatoid arthritis (RA) pathogenesis by investigating the presence of B19V infection markers (genomic sequences and virus-specific antibodies) in association with the level of cytokines and RA clinical activity and aggressiveness. A total of 118 RA patients and 49 age- and sex-matched healthy volunteers were enrolled in the study. Nested PCR was used to detect B19V sequences in whole blood and cell-free plasma DNA, ELISA to detect virus-specific antibodies and cytokine levels in plasma and recomLine dot blot assay for antibodies to separate B19V antigens. The detection frequency of B19V DNA was higher in patients with RA (25.4 %) in comparison with healthy persons (18.4 %). B19V DNA in cell-free plasma (B19^+^p) was detected significantly often in RA patients in comparison with healthy controls (13.6 vs 2 %; *P*=0.0002). RA B19^+^p patients had higher disease activity and aggressiveness, decreased haemoglobin and increased erythrocyte sedimentation rates. IL-6 plasma levels were significantly higher in RA patients than in controls. Within the RA patients’ group the IL-6 level was significantly increased in B19^+^p patients with disease activity scores of DAS28>5.2, high C-reactive protein and low haemoglobin. Contrary to the healthy controls, the majority of RA B19^+^p patients did not have antibodies to VP-1S (VP1u) and VP-N (N-terminal half of structural proteins VP1 and VP2), which correspond to the epitopes of neutralizing antibodies. These results indicate that B19V infection at least in some patients is involved in RA pathogenesis.

## Introduction

Human parvovirus B19 (B19V) is a small DNA virus with a worldwide distribution. The genome of B19V consists of single-stranded 5.6 kb DNA that encodes three large proteins. The non-structural protein NS1 is composed of 671 aa. Structural capsid proteins VP1 and VP2 are encoded in the same ORF with production of proteins of 84 and 58 kDa, respectively. VP1 and VP2 are identical, with the exception of 227 aa at the amino-terminal end of the VP1 protein, the so-called VP1-unique region (VP1u) (Cotmore *et al.*, 1986; Ozawa & Young, 1987; Ozawa *et al.*, 1987). B19V is normally transmitted through the respiratory route, but can also be transmitted from the mother to the foetus (Berry *et al.*, 1992; Brown *et al.*, 1984; Clewley *et al.*, 1987; Kinney *et al.*, 1988; Musiani *et al.*, 1999), through bone marrow (Cohen *et al.*, 1997; Heegaard & Laub Petersen, 2000; Ozawa & Young, 1987) and organ transplantations (Bertoni *et al.*, 1997; Lui *et al.*, 2001), and via transfused blood products (Juhl *et al.*, 2014). B19V replicates in red blood cell precursors in the bone marrow, however, it could persist in other cells (Bratslavska *et al.*, 2015; Millá *et al.*, 1993; Morey & Fleming, 1992; Söderlund-Venermo *et al.*, 2002). B19V DNA was found in peripheral blood and synovial fluid cells (Kozireva *et al.*, 2008), and VP1 protein was detected in synovial cells including lymphocytes, macrophages and neutrophils (Takahashi *et al.*, 1998). Although antibodies to conformational and linear epitopes of VP1 and VP2 capsid proteins were detected (Azzi *et al.*, 2004; Corcoran *et al.*, 2000; Manaresi *et al.*, 1999, 2004; Söderlund *et al.*, 1995), the most important are neutralizing antibodies directed to the VP1u and VP1–VP2 junction regions (Saikawa *et al.*, 1993; Zuffi *et al.*, 2001).

B19V is associated with many clinical disorders. This virus causes erythema infectiosum (fifth disease) in children. B19V infection can be asymptomatic or can cause various complications such as arthralgia, arthritis, transient aplastic crisis and chronic anaemia in adults (Chorba *et al.*, 1986; Dijkmans *et al.*, 1988; Lefrère *et al.*, 1986; Reid *et al.*, 1985; White *et al.*, 1985). There is some evidence that B19V infection is more frequent in patients with certain autoimmune diseases [juvenile rheumatoid arthritis (RA) (Oğuz *et al.*, 2002), systemic lupus erythematosus (Hsu & Tsay, 2001), Sjögren's syndrome (Ramos-Casals *et al.*, 2000), thyroiditis (Lehmann *et al.*, 2008; Wang *et al.*, 2010) and chronic fatigue syndrome (Chapenko *et al.*, 2012)] and can even play a role in pathogenesis. However, some publications suggesting that B19V does not play any role in the pathogenesis, but may present a clinical and serological tableau making it difficult to distinguish between a viral infection and systemic lupus erythematosus (Moore *et al.*, 1999; Sève *et al.*, 2005). Some authors did not find a link between B19V infection and Sjögren's syndrome (De Re *et al.*, 2002; De Stefano *et al.*, 2003) or juvenile arthritis (Söderlund *et al.*, 1997). B19V infection is often associated with chronic arthritis, although aetiologic associations with RA are conflicting (Kerr, 2000; Takahashi *et al.*, 1998).

RA is one of the most common inflammatory autoimmune diseases characterized by persistent synovitis, systemic inflammation and production of autoantibodies (Scott *et al.*, 2010). The molecular mechanisms of RA pathogenesis are not fully understood. It is believed that approximately half of the risk factors for RA are attributable to genetic factors such as the HLA alleles, while the other half of the risks are environmental factors including infection and smoking (Arleevskaya *et al.*, 2014; McInnes & Schett, 2011; Okada *et al.*, 2014). Clinical and animal model studies have suggested that infections by many micro-organisms, such as *Porphyromonas gingivalis* (Bartold *et al.*, 2010; Hitchon *et al.*, 2010), *Proteus mirabilis* (Arabski *et al.*, 2012), Epstein–Barr virus (Mourgues *et al.*, 2016) and mycoplasma contribute to the aetiopathogenesis of RA (Durigon *et al.*, 1993). Higher rates of serological evidence of infections with Epstein–Barr virus and cytomegalovirus were detected in RA patients than in population controls (Magnusson *et al.*, 2010; Meron *et al.*, 2010).

The aim of the present study was to clarify the possible involvement of B19V infection in RA pathogenesis by investigating the presence of B19V infection markers in association with RA clinical activity and the level of cytokines.

## Results

### Frequency of B19V DNA in RA patients and healthy controls

In this study we investigated the frequency of B19V infection markers in the blood of RA patients. The B19V genomic DNA was determined in whole blood and cell-free plasma (Table 1). Persons in whose blood or plasma B19V DNA was detected were named B19^+^. If virus DNA was not detected, such persons were named B19^−^. The mean age of B19^+^ and B19^−^ RA patients and control groups’ individuals was 58.3±13.6 and 58.3±13.0 years and 55.3±9.0 and 49.2±11.3 years, respectively. The frequency of B19V DNA was higher in patients with RA (30/118; 25.4 %) in comparison with control group individuals (9/49; 18.4 %). The same tendency was observed in both males [6/19 (31.6 %) of RA patients and 2/12 (16.7 %) of controls were B19V DNA positive] and females [24/99 (24.2 %) of RA patients and 7/37 (18.9 %) of controls were B19V DNA positive]. B19V DNA was detected in whole blood (B19^+^b), cell-free plasma (B19^+^p) or both whole blood and plasma (B19^+^b/p). Table 1 shows the localization of virus DNA in all investigated groups. In RA patients B19V DNA was mostly found in the cell-free plasma (53 % from all B19^+^), while in the control group the virus DNA was found mostly in the whole blood (89 % from all B19^+^). Our results showed that the frequency of B19V latent/persistent (B19V sequences presented only in DNA extracted from the whole blood) and active (B19V sequences presented in DNA extracted from the cell-free blood plasma) infection markers is different in RA patients and healthy people (Table S1, available in the online Supplementary Material). We investigated how the localization of virus DNA (blood and plasma, blood or plasma) correlates with B19V seroprevalence and antibody spectrum, the level of cytokines in plasma and disease clinical activity. Table 2 shows the clinical characteristics [white blood cells (WBC), haemoglobin (HgB), erythrocyte sedimentation rate (ESR), C-reactive protein (CRP), cyclic citrullinated peptide antibody (anti-CCP), disease activity score (DAS28), disease duration and rheumatoid factor (RF) result] of RA patients according to virus DNA localization. RA patients (68.8 %) from the B19^+^p group were strongly positive (>60 EU ml^−1^) for anti-CCPs, while only 25 % of B19^+^b/p, 33.3 % of B19^+^b and 48.9 % of B19^−^ patients had anti-CCP levels >60 EU ml^−1^ (Table 2). Also, 68.8 % of RA patients from the B19^+^p group had high disease activity scores (DAS28>5.2). There were no more than 50 % of patients with DAS28>5.2 in the groups B19^+^b, B19^+^b/p and B19^−^. A higher percentage of patients from the RA B19^+^p group had decreased HgB (68.8 %) and increased ESR (87.5 %), in comparison with persons in the other groups. The presence of B19V DNA in RA patients did not influence the expression of RF, disease duration or CRP level (Table 2).

**Table 1.** Presence of B19V genomic sequence in the blood DNA of RA patients and healthy persons The values marked by stars are significant according to Fisher’s exact tests: *P<0.05, **P<0.0005. B19V, Parvovirus B19; B19^+^, parvovirus B19 DNA positive; B19^−^, parvovirus B19 DNA negative; RA, rheumatoid arthritis; Contr., healthy persons.

Investigated persons (total)		Age±sd (years)	B19^+^ samples								B19^−^ samples	
			Whole blood		Cell-free blood plasma		Whole blood and cell-free blood plasma		All positive		N	% Total
			N	% Total	N	% Total	N	% Total	N	% Total		
Females												
Contr.	37	50.3±10.4	6	16.2	1	2.7	0	0	7	18.92	30	81.08
RA	99	57.3±13.0	6	6.1	12	12.1	6	6.1	24	24.24	75	75.76
Males												
Contr.	12	49.8±13.8	2	16.66	0	0	0	0	2	16.66	10	83.33
RA	19	63.5±12.7	0	0	4	21.1	2	10.5	6	31.58	13	68.42
All investigated (males+females)												
Contr.	49	50.2±11.3	8	16.3**	1	2.0*	0	0	9	18.37	40	81.63
RA	118	58.3±13.0	6	5.1**	16	13.6*	8	6.8	30	25.42	88	75.58

**Table 2.** Clinical characteristics of rheumatoid arthritis patients Table 2 shows the number of patients with normal values (norm.), higher than normal (>norm.), lower than normal (<norm.) or with appropriate indicated values. The percentage of patients with indicated parameters are shown. WBC, White blood cells; HgB, haemoglobin; ESR, erythrocyte sedimentation rate; CRP, C-reactive protein; anti-CCP, cyclic citrullinated peptide antibody; DAS28, disease activity score; RF, rheumatoid factor.

Clinical data		Parvovirus B19 DNA positive in			Parvovirus B19 DNA negative
		Blood and plasma	Blood	Plasma	
WBC (×10^9^ l^−1^)	Norm.	5	3	11	46
	>Norm.	3	3	5	42
	**% >Norm.**	**37.5**	**50.0**	**31.3**	**47.7**
HgB (g l^−1^)	Norm.	3	4	5	48
	<Norm.	5	2	11	40
	**% <Norm.**	**62.5**	**33.3**	**68.8**	**45.5**
ESR (mm h^−1^)	Norm.	3	3	2	19
	>Norm.	5	3	14	68
	**% >Norm.**	**62.5**	**50.0**	**87.5**	**77.3**
CRP (mg l^−1^)	Norm.	3	2	5	22
	>Norm.	5	4	11	66
	**% >Norm.**	**62.5**	**66.7**	**68.8**	**75.0**
Anti-CCP (EU ml^−1^)	1–60	6	4	5	45
	>60	2	2	11	43
	**% >60**	**25.0**	**33.3**	**68.8**	**48.9**
DAS28	<5.2	4	5	5	43
	>5.2	4	1	11	43
	**% >5.2**	**50.0**	**16.7**	**68.8**	**50.0**
Disease duration (years)	0–10	5	2	8	44
	>10	3	4	8	44
	**% >10**	**37.5**	**66.7**	**50.0**	**50.0**
RF (U ml^−1^)	1–100	3	3	9	50
	>100	5	3	7	38
	**% >100**	**62.5**	**50.0**	**43.8**	**44.0**

### Antibodies to B19V antigens in the plasma of RA patients and healthy controls

Antibodies to the main capsid protein VP2 were determined in the plasma of all investigated (118 RA and 49 controls) persons using the Biotrin ELISA kit (Tables 3 and S2). The percentages of RA and control persons carrying antibodies to VP-2P, VPN, VP-1S (VP1u), VP-2r, VP-C and NS-1 are shown in Table 3. The individual raw data are shown in Table S2. Table 3 shows that the majority (78–90 %) of subjects (healthy controls and RA patients, B19V DNA positive or negative) had IgG class antibodies to VP2 protein; however they did not have IgM class antibodies. Part of the plasma samples (55 from RA patients and 28 from healthy persons) were analysed using the Mikrogen Diagnostik's recomLine dot blot kit. According to manufacturer’s (Mikrogen Diagnostik) manual, recomLine dot blot enables determination of antibodies to the various virus antigens: VP-2P (main capsid antigen, conformation epitope), VP-N (the N-terminal half of the structural proteins VP1 and VP2), VP-1S [corresponds to the unique region of VP1 (VP1u)], VP-2r (main capsid antigen, linear epitope), VP-C (the C-terminal half of the structural proteins VP1 and VP2) and NS-1 (non-structural protein) (Pfrepper *et al.*, 2005). VP-2P protein in recomLine dot blot corresponds to the VP2 protein in Biotrin ELISA. Although IgG ELISA seems to be more sensitive [77 samples out of 83 were positive in IgG VP2 ELISA, whereas only 59 samples out of 83 were positive in IgG VP-2P dot blot (Table S2)], the results obtained using Biotrin VP2 IgG ELISA and recomLine with IgG VP-2P dot blot are comparable according to the Spearman's correlation test (*r*=0.909, *P*<0.0001). The IgG class antibodies to VP2/VP-2P proteins were detected in the plasma of majority of RA patients’ and controls’ plasma samples (to VP2 in 91 % of RA B19^+^, 94 % of RA B19^−^, 79 % of B19^+^ controls and 100 % of B19^−^ controls; to VP-2P in 77 % of RA B19^+^, 67 % of RA B19^−^, 78 % of B19^+^ controls and 68 % of B19^−^ controls). The seroprevalence of IgG antibodies to VP2/VP-2P did not differ significantly between the groups of RA patients and healthy controls. However, the seroprevalence of IgG antibodies to VP-N and VP-1S was different between RA B19^+^b and RA B19^+^p patients. In the RA B19^+^b group, 80 % of patients had IgG antibodies to VP-N and VP-1S, while in the RA B19^+^p group, only 46 and 38 % of patients had antibodies to VP-N and VP-1S, respectively. Only a limited number of persons had IgG antibodies to VP-2r (five RA B19^+^, five RA B19^−^ and three B19^−^ controls), VP-C (one RA B19^−^ and one B19^−^ control) and NS-1 (two B19^−^ controls) (see Tables 3 and S2). Although we did not detect IgM antibodies to VP2 protein by ELISA in the plasma of RA patients and healthy controls, recomLine dot blot assay showed the presence of IgM class antibodies in some RA patients. IgM antibodies to VP-2P were detected in the plasma of 11, to VP-N in 7 and to VP-1S and VP-2r in 2 RA patients (Table S2). IgM antibodies to the proteins mentioned were not detected in the plasma of healthy controls (Tables 3 and S1). The presence of IgM class antibodies in RA did not show an association with the presence of B19 DNA in plasma or whole blood.

**Table 3.** B19V seroprevalence among RA patients and healthy persons Percentage of persons with antibodies to parvovirus B19 VP2 protein (detected using Biotrin ELISA kit) and to various parvovirus B19 antigens (detected using Mikrogen recomLine kit). B19^+^ RA patients were divided into the groups according to the viral DNA findings: in blood and cell-free blood plasma (b/p), blood (b) and cell-free blood plasma (p). *N*, Number of persons; B19^+^, parvovirus B19 DNA positive; B19^−^, parvovirus B19 DNA negative; Contr., healthy controls.

	All investigated samples			Samples tested by recomLine dot blot														
	*N*	Positive in VP2 ELISA		*N*	Positive in VP2 ELISA		Positive in recomLine dot blot											
							VP-2P		VP-N		VP-1S		VP-2r		VP-C		NS-1	
		*N*	%		*N*	%	*N*	%	*N*	%	*N*	%	*N*	%	*N*	%	*N*	%
IgG antibodies to B19V proteins																		
RA B19^+^b/p	8	7	87.5	4	3	75.0	3	75.0	2	50.0	2	50.0	2	50.0	0	0	0	0
RA B19^+^b	6	5	83.3	5	5	100	5	100	4	80.0	4	80.0	1	20.0	0	0	0	0
RA B19^+^p	16	15	93.8	13	12	92.3	9	69.2	6	46.2	5	38.5	2	15.4	0	0	0	0
RA B19^+^	30	27	90.0	22	20	90.9	17	77.3	12	54.5	11	50.0	5	22.7	0	0	0	0
RA B19^−^	88	76	86.4	33	31	93.9	22	66.7	18	54.5	14	42.4	5	15.2	1	3.0	1	3.0
Contr. B19^+^	9	7	77.8	9	7	77.8	7	77.8	6	66.7	6	66.7	0	0	0	0	0	0
Contr. B19^−^	44	36	81.8	19	19	100	13	68.4	11	57.9	11	57.9	3	15.8	1	5.3	2	10.5
IgM antibodies to B19V proteins																		
RA B19^+^b/p	8	0	0	4	0	0	2	50.0	2	50.0	0	0	0	0	0	0	0	0
RA B19^+^b	6	0	0	5	0	0	2	40.0	0	0	0	0	1	20.0	0	0	0	0
RA B19^+^p	16	0	0	9	0	0	2	22.2	3	33.3	2	22.2	0	0	0	0	0	0
RA B19^+^	30	0	0	18	0	0	6	33.3	5	27.8	2	11.1	1	5.6	0	0	0	0
RA B19^−^	88	0	0	12	0	0	5	41.7	2	16.7	0	0	1	8.3	0	0	0	0
Contr. B19^+^	9	0	0	9	0	0	0	0	0	0	0	0	0	0	0	0	0	0
Contr. B19^−^	44	0	0	11	0	0	0	0	0	0	0	0	0	0	0	0	0	0

### Levels of IL-6 in the plasma of RA patients and healthy controls

**Fig. 1.**
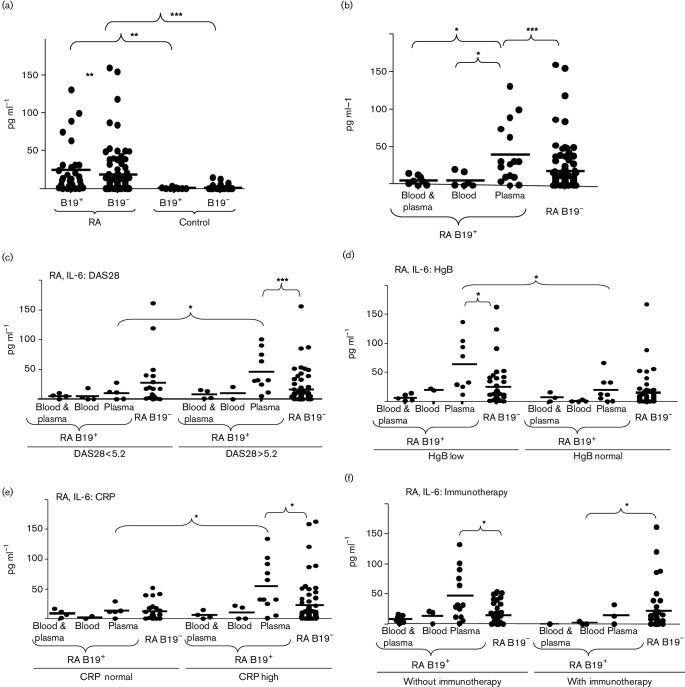
Amount of IL-6 in the plasma of parvovirus B19 DNA positive (B19^+^) and parvovirus B19 DNA negative (B19^−^) persons. IL-6 in the plasma of rheumatoid arthritis (RA) patients and healthy age-matched persons (control) (a). IL-6 in the plasma of RA patients, divided according to the localization of virus DNA (b). RA patients are divided into the groups according to the findings of virus DNA (in blood, in cell-free blood plasma and in both blood and cell-free plasma) and DAS28<5.2 and DAS28>5.2 (c); HgB lower than normal and HgB normal (d); normal values of CRP and CRP high (e) and patients with and without immunotherapy (f). One symbol shows the amount of IL-6 (pg ml^−1^) of one person. Results are significant according to Mann–Whitney *U* test: *P<0.05, **P<0.005 or ***P<0.0005.

The levels of IL-6 were determined in the blood plasma of all investigated RA patients and controls. The levels of IL-6 were significantly higher in the plasma of RA patients (25.0±33.6 pg ml^−1^ in B19^+^ group and 19.0±30.5 pg ml^−1^ in B19^−^ group) in comparison with the persons in the control group (0.8±1.0 pg ml^−1^ in B19^+^ group and 1.6±3.4 pg ml^−1^ in B19^−^ group) (Fig. 1a). There were no significant differences between the plasma level of IL-6 in B19^+^ and B19^−^ controls. However, RA B19^+^ patients had significantly higher average levels of IL-6 than RA B19^−^ patients. Importantly, we found that the IL-6 levels were particularly increased in RA B19^+^p patients’ group (40.6±39.7 pg ml^−1^) (Fig. 1b). The levels of IL-6 in patients from the RA B19^+^b and RA B19^+^b/p groups were 7.2±9.8 and 7.0±5.9 pg ml^−1^, respectively. Our data showed that some clinical data (anti-CCP, DAS28, HgB and ESR) differ when B19V DNA was detected in the plasma (Table 2). Therefore, the cytokine levels were analysed according to the findings in respect of clinical parameters. The levels of IL-6 in the plasma of RA B19^+^ patients were investigated according to RA disease activity. The patients were divided according to DAS28 in two groups: DAS28<5.2 (low disease activity or remission) and DAS28>5.2 (high disease activity) (Fig. 1c). Fig. 1(c) shows that the level of IL-6 is increased in RA B19^+^p patients with DAS28>5.2 (IL-6 54.4±40.7 pg ml^−1^), but not in patients with DAS28<5.2 (IL-6 10.6±11.4 pg ml^−1^). Also, the RA patients were divided into groups according to HgB levels. The levels of IL-6 were similarly low in all B19^+^ and B19^−^ RA patients' groups with normal values of HgB. However, the levels of IL-6 were increased in RA B19^+^p patients with lower values of HgB. IL-6 concentration in the plasma of RA B19^+^p patients was 61.6±43.6 pg ml^−1^ when the level of HgB was low and 19.7±21.8 pg ml^−1^ when the level of HgB was normal (Fig. 1d). We investigated IL-6 levels in RA patients according to CRP level (Fig. 1e). The IL-6 concentration in the plasma was lower in B19^+^p RA patients with normal levels of CRP (IL-6 12.7±10.0 pg ml^−1^). RA B19^+^p patients with increased CRP levels had increased IL-6 concentrations (IL-6 53.4±41.9 pg ml^−1^) in comparison with RA B19^+^b/p, B19^+^b and B19^−^ patients' groups (the average IL-6 concentration varied from 1.8 to 12 pg ml^−1^). The concentrations of IL-6 were significantly higher also in RA B19^+^ and RA B19^−^patients' groups that fulfilled the following criteria: low HgB, high ESR, DAS28>5.2 in comparison to RA B19^+^ and RA B19^−^patients' groups that have normal HgB, normal ESR and DAS28<5.2 (Fig. S1). The IL-6 concentration in the plasma was also analysed taking into account the method of treatment. The concentration of IL-6 was higher (46.6±41.5 pg ml^−1^) in RA B19^+^p patients without immunotherapy. However, RA B19^+^p patients after immunotherapy had lower (14.8±16.0 pg ml^−1^) levels of IL-6. IL-6 plasma levels in RA B19^+^b and RA B19^+^b/p patients were low, whether or not they had had immunotherapy (up to 14 pg ml^−1^) (Fig. 1f). The patients were divided into different groups with low, normal or high values of WBC, ESR, anti-CCP and RF. The level of IL-6 was increased in the RA B19^+^p patients group, but we did not observe significant differences in the concentration of IL-6 by comparing B19^+^p RA patients with normal or increased/decreased values of WBC, ESR, anti-CCP and RF (Fig. S2). Also, we did not find significant changes in IL-2, IL-4, IL-10, IL-12, IL-17, TNF-α and IFN-γ levels in the plasma of RA B19^+^p patients (Table S3). Next, we analysed the correlation between cytokine concentrations in the plasma and clinical parameters according to the B19V DNA localization (Table S4). We observed some significant correlations that were characteristic only of patients from RA B19^+^p group. There were positive correlations between IL-2 and IL-12 levels (*r*=0.91, *P*<0.0005), IL-10 and IL-12 levels (*r*=0.71, *P*<0.005), IL-12 level and disease duration (*r*=0.54, *P*<0.05), IL-12 level and DAS28 (*r*=0.53, *P*<0.05), IL-10 level and CRP concentration (*r*=0.6, *P*<0.05), IL-6 level and CRP concentration (*r*=0.7, *P*<0.005) and IFN-γ level and CRP concentration (*r*=0.58, *P*<0.05). Negative correlations were observed between IL-4 and HgB levels (*r*=−0.5, *P*<0.05) and IL-6 and HgB levels (*r*=−0.69, *P*<0.005). Such correlations were not observed when B19V DNA was detected in blood and plasma, or blood or not detected at all.

We calculated correlations between amount of antibodies and levels of cytokines for various indices of clinical data. We found significant negative correlations between the amount of antibodies to VP-C and levels of IL-6 in plasma (*r*=−0.6, *P*<0.05), between antibodies to VP-N, VP-1S, VP-2r and age (*r*=−0.6, *P*<0.05) and between antibodies to VP-2r and RF (*r*=−0.59, *P*<0.5). A significant positive correlation was noticed between levels of antibodies to VP2 and levels of IFN-γ (*r*=0.59, *P*<0.5). Such correlations were observed only in RA B19^+^p patients and were not noticeable in the groups of RA B19^+^b, RA B19^+^b/p, RA B19^−^, control B19^+^ and control B19^−^ persons.

## Discussion

B19V infection in RA patients was investigated in this study. Our results show that the presence of B19V DNA is more frequent in RA patients than in age- and sex-matched people that do not have RA and do not suffer from the pain in the joints. In addition, in contrast to persons in the control group where B19V DNA is found mostly in whole blood (persistent infection in latent phase), the majority of B19V DNA-positive RA patients have virus DNA in cell-free blood plasma, i.e. they have persistent infection in the active phase. Our data coincide with previously published data, in showing that the prevalence of B19V DNA in serum, plasma or peripheral blood cells was significantly higher in patients with RA than in controls (Caliskan *et al.*, 2005; Chen *et al.*, 2006; Kozireva *et al.*, 2008; Tzang *et al.*, 2009b) and that RA activity was higher in patients with latent/persistent B19V infection (Kakurina *et al.*, 2015). However, some authors concluded that B19V is not involved in the aetiopathogenesis of RA (Kerr *et al.*, 1995; Nikkari *et al.*, 1995). Paradoxically, we find that B19V DNA is present only in the cell-free blood plasma of some RA patients but not in their whole blood which also includes plasma. This could be due to low copies of virus DNA in the plasma. When DNA is isolated from the cell-free plasma, viral DNA is concentrated and could be detectable. However, when DNA is purified from whole blood, the majority of the DNA comes from blood cells, and the viral DNA (present in plasma) is diluted and is not detectable due to low copies and insufficient PCR sensitivity. Therefore, it is most likely that the B19V DNA detected in whole blood represents viral DNA from B19V containing blood cells. Moreover, the presence of B19V DNA in peripheral blood cells has been shown previously by Kakurina *et al.* (2015), while the presence of VP1 protein in synovial cells including lymphocytes, macrophages and neutrophils (e.g. cells present in the blood) was shown by Takahashi *et al.* (1998).

Since B19V DNA in RA patients dominates in the plasma, we analysed how it affects the clinical data, the antibody response to various virus proteins and the levels of cytokine expression in the blood. Our data show that RA patients with B19V DNA in cell-free plasma have also higher levels of anti-CCP and higher scores of DAS28, indicating higher disease aggressiveness and activity, respectively. Many of the RA patients with B19V DNA sequences in plasma DNA also have decreased HgB (68.8 %) and increased ESR (87.5 %), in comparison with the RA patients who have virus sequences in whole blood DNA or do not have it at all. This is consistent with previously known data that persistent B19V infection in humans may cause chronic anaemia (Kurtzman *et al.*, 1989) and, as our data suggest, that B19V may play a certain role in the pathogenesis of RA.

Although the majority of RA patients in our study have IgG class B19V-specific antibodies, the spectrum of antibodies varies. A smaller percentage of RA B19^+^p patients, compared with healthy controls or RA B19^+^b patients, have VP-1S- and VP-2P-specific antibodies. VP-1S and VP-N antigens overlap with the unique region of VP1. It is known that the epitopes of neutralizing antibodies are located within the VP1u and VP1–VP2 junction regions (Saikawa *et al.*, 1993), and also within the amino-terminal portion of VP2 (Sato *et al.*, 1991). Neutralizing antibodies are produced shortly after infection (von Poblotzki *et al.*, 1995) and elicit a long-lasting immune response (Zuffi *et al.*, 2001). The production of neutralizing antibodies to VP1u is very important for immune protection against viral infection, while VP1u is essential for B19V binding and internalization (Leisi *et al.*, 2013). In our study, the lack of neutralizing antibodies in RA B19^+^p patients could explain the higher frequency of B19V persistent infection in the active phase or viraemia (cell-free virus in the blood stream), because the production of neutralizing antibody to capsid protein in persistently infected subjects plays a major role in limiting B19V infection in human (Kurtzman *et al.*, 1989). B19^+^p RA patients differ from other (B19^+^b/p, B19^+^b and B19^−^) RA patients and from healthy controls, in that not only do the majority of them not have antibodies to VP-1S and VP-N, but also in that the amount of antibodies to VP-N, VP-1S and VP-2r negatively correlates with age, the amount of antibodies to VP-2r negatively correlates with RF, the amount of antibodies to VP-C negatively correlates with levels of IL-6 and HgB values negatively correlate with levels of IL-4 and IL-6. Antibodies to VP-2P and VP2 positively correlate with WBC and IFN-γ, respectively. Positive correlations were observed between the levels of IL-2 and IL-12, IL-10 and IL-12, IL-12 and disease duration, IL-12 and DAS28, IL-10 and CRP, IL-6 and CRP and IFN-γ and CRP. Such correlations were not observed in other groups of RA patients and healthy persons. The presence of these correlations, in the RA B19^+^p patient group only, also indicates that B19V plays a role in RA pathogenesis.

Our data and previous (Manicourt *et al.*, 1993; Stabler *et al.*, 2004) data show that levels of IL-6 are increased in the serum of patients with RA compared with controls. Our data indicate significantly increased IL-6 concentrations in the plasma of B19V-infected RA patients, but only in those who have a virus genomic sequence in cell-free plasma DNA. The levels of IL-6 in RA B19^+^b and RA B19^+^b/p patients are even lower than in RA B19^−^ patients, and are similar to levels in the plasma of healthy controls. Also, IL-6 levels are increased in RA patients with DAS28>5.2, low HgB and high CRP and in those not receiving immunotherapy.

Serum IL-6 level as a potential biomarker reflecting RA disease activity is described previously (Shimamoto *et al.*, 2013). B19V infection also could cause the increased IL-6 concentration in blood. Increased levels of IL-6 mRNA or increased serum IL-6 levels are detected during acute B19 infection, but not by follow-up (Kerr *et al.*, 2001,^2004^). The levels of IL-6 are raised in B19-infected patients with chronic fatigue syndrome (Chapenko *et al.*, 2012).

It is shown that B19V proteins NS1 (Hsu *et al.*, 2006; Kerr *et al.*, 2001) and VP1u (Tzang *et al.*, 2009a) induce IL-6 mRNA expression. B19V proteins during viraemia could induce IL-6 production in RA patients. Takahashi *et al.* (1998) have shown *in vitro* that B19V induced IL-6 production could be suppressed by the addition of neutralizing anti-VP1 antibody. However, the majority of RA patients do not have neutralizing antibodies to the VP1 N-terminal part, and this could be a reason for B19V infection activity and increased levels of IL-6 in blood. The active phase of persistent B19V infection in RA patients is associated with increased disease activity, an increased amount of anti-CCP, decreased HgB and increased ESR. In summary, our study suggests that B19V infection, at least in some patients, plays a role in pathogenesis of RA.

## Methods

### Blood samples of patients.

A total of 118 patients with RA (99 females and 19 males, mean age 58.3±13.0 years) and 49 age- and sex-matched healthy volunteers (37 females and 14 males, mean age 50.2±11.3 years) as the control group were enrolled in this study. Participants in the study were selected from patients seen at the Vilnius University Hospital Santariskiu Clinics. RA was diagnosed according to 2010 ACR/EULAR (American College of Rheumatology/ European League Against Rheumatism) RA Classification Criteria for the classification of RA by expert rheumatologists (Cotmore *et al.*, 1986). We did not include chronic (over 6 months) B19V-induced polyarthritis with transient RF in this study as RA. Also, reactive arthritis induced by parvovirus infection does not fulfil the RA Classification Criteria for the classification of RA. RA group patients were treated by immunosupressants. Some of the RA patients have comorbidities (e.g. cardiovascular diseases). All patients underwent an extensive medical examination, whole blood analysis and extensive serologic evaluation which included tests for WBC, HgB, ESR, CRP, anti-CCP, DAS28 and RF. Blood samples were collected in vacutainers with K_2_EDTA and processed within 4 h of collection. The whole blood samples at aliquots of 0.5 ml and the plasma samples at aliquots of 0.2 ml were frozen and stored at −80 °C until used for analysis.

This study was approved by the Vilnius Regional Biomedical Research Ethics Committee.

### DNA extraction and PCRs.

DNA was isolated from 0.5 ml of the whole blood and from 0.2 ml of cell-free plasma. The sample was digested by proteinase K and extracted by a standard phenol–chloroform technique. B19V genomic DNA was detected by a nested PCR. The sequences of B19V primers were as described by Durigon *et al.* (1993). The sequences of the primers were: F**-**out AATACACTGTGGTTTTATGGGCCG, R-out CCATTGCTGGTTATAACCACAGGT; F-in GAAAACTTTCCATTTAATGATGTAG, R-in CTAAAATGGCTTTTGCAGCTTCTAC. The PCR was performed using Maxima Hot Start Polymerase (Thermo Scientific) according to the manufacturer’s recommendations. Positive and negative (DNA without B19V genomic sequences) controls were included in every PCR as well as water controls after every third sample. The cycling conditions of the first reaction were: 95 °C 10 min, 40 cycles: 95 °C 45 s, 55 °C 45 s, 75 °C 1 min and elongation 75 °C 2 min. Two microlitres of the product from first PCR was subjected to the second reaction of PCR. The cycling conditions of the second reaction were the following: 95 °C 10 min, 40 cycles: 95 °C 45 s, 56 °C 45 s, 75 °C 45 s and elongation 75 °C 2 min. The PCR products (284 bp) were analysed in 3 % agarose gel.

### Detection of antibodies to B19V antigens.

IgM and IgG antibodies to B19V antigens were detected in blood plasma. Antibodies to VP2 protein were detected using Parvovirus B19 IgM and IgG Enzyme Immunoassay kits (Biotrin). The assays were performed and the results were calculated according to the manufacturer's instructions. Data comparison between different assay runs was facilitated by using an index value. The index was calculated as the ratio of the sample’s optical density (or OD_450 nm_) measurements to the cutoff’s OD_450 nm_. An index value <0.9 or >1.1 indicated sample negativity or positivity, respectively. Equivocality was indicated if the index value was in the range 0.9–1.1.

The antibodies to various virus proteins were determined using recomLine Parvovirus B19 IgG and IgM kits (Mikrogen). IgM and IgG class antibodies to VP-2P (main capsid antigen, conformation epitope), VP-N (N-terminal half of the structural proteins VP1 and VP2), VP-1S (VP1u), VP-2r (main capsid antigen, linear epitope), VP-C (C-terminal half of the structural proteins VP1 and VP2) and NS-1 (non-structural protein) were determined. The assays were performed according to the manufacturer’s instructions. The bands of the blots were scanned and the band density was quantified using ImageJ 1.49 software.

### Determination of the cytokine concentration in the plasma.

The IL-2, IL-4, IL-6, IL-10, IL-12, IL-17 and TNF-α levels in the plasma were detected using human IL-2, IL-4, Il-6, IL-10, IL-12 (p70), IL-17 and TNF-α ELISA MAX Standard Sets (BioLegend) according to the manufacturer’s recommendations. The IFN-γ level was detected by two-site ELISA using home-made murine mAbs to human IFN-γ and recombinant human IFN-γ (Life Technologies), as the standard as described before (Voll *et al.*, 1997). The plasma for ELISA was diluted 1 : 3. The results were calculated using ‘Gen5’ software.

### Statistical analysis.

Fisher's exact test was used to calculate rates and proportions between the groups. Mann–Whitney *U* test was used to compare two groups of patients with non-normal distribution of data. The Spearman’s rank correlation coefficient was used to discover the strength of correlation between two sets of data. *P*<0.05 was considered significant.
