# Supplementary material for: Frequency and significance of parvovirus B19 infection in patients with rheumatoid arthritis
Source: J Gen Virol. 2016 Dec 15;97(12):3302–12. doi: 10.1099/jgv.0.000621 (PMC5203673; doi:10.1099/jgv.0.000621)
Supplement: Supplementary File 1 [file jgv-97-3302-s001.pdf]

Fig. S1

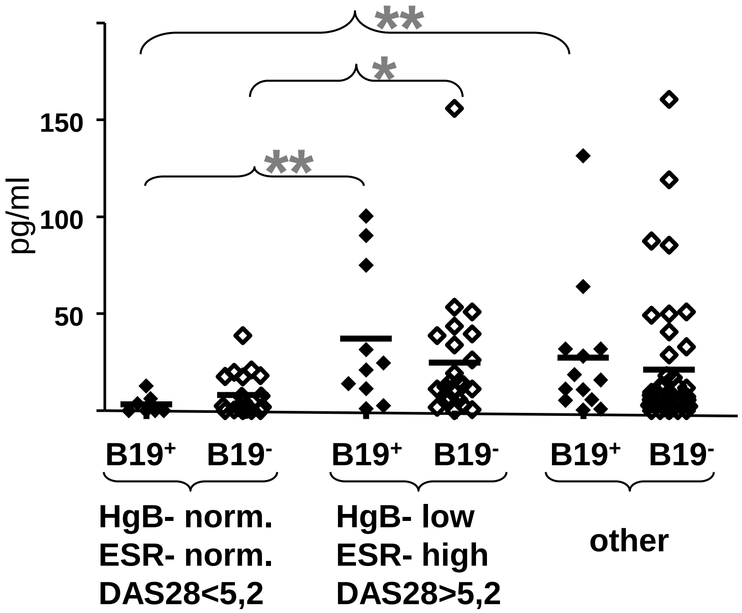

Fig. S1. The levels of IL-6 in B19 DNA positive ($B19^{+}$) and parvovirus B19 negative ($B19^{-}$) rheumatoid arthritis patients that fulfill the criteria: low HgB, high ESR, DAS28>5.2 and normal HgB, normal ESR and DAS28<5.2. One symbol shows the amount of IL-6 (pg /mL) of one person. The results significant according to Mann-Whitney U test are marked by stars: p<0.05 (*) or p<0.005 (**)

Fig. S2

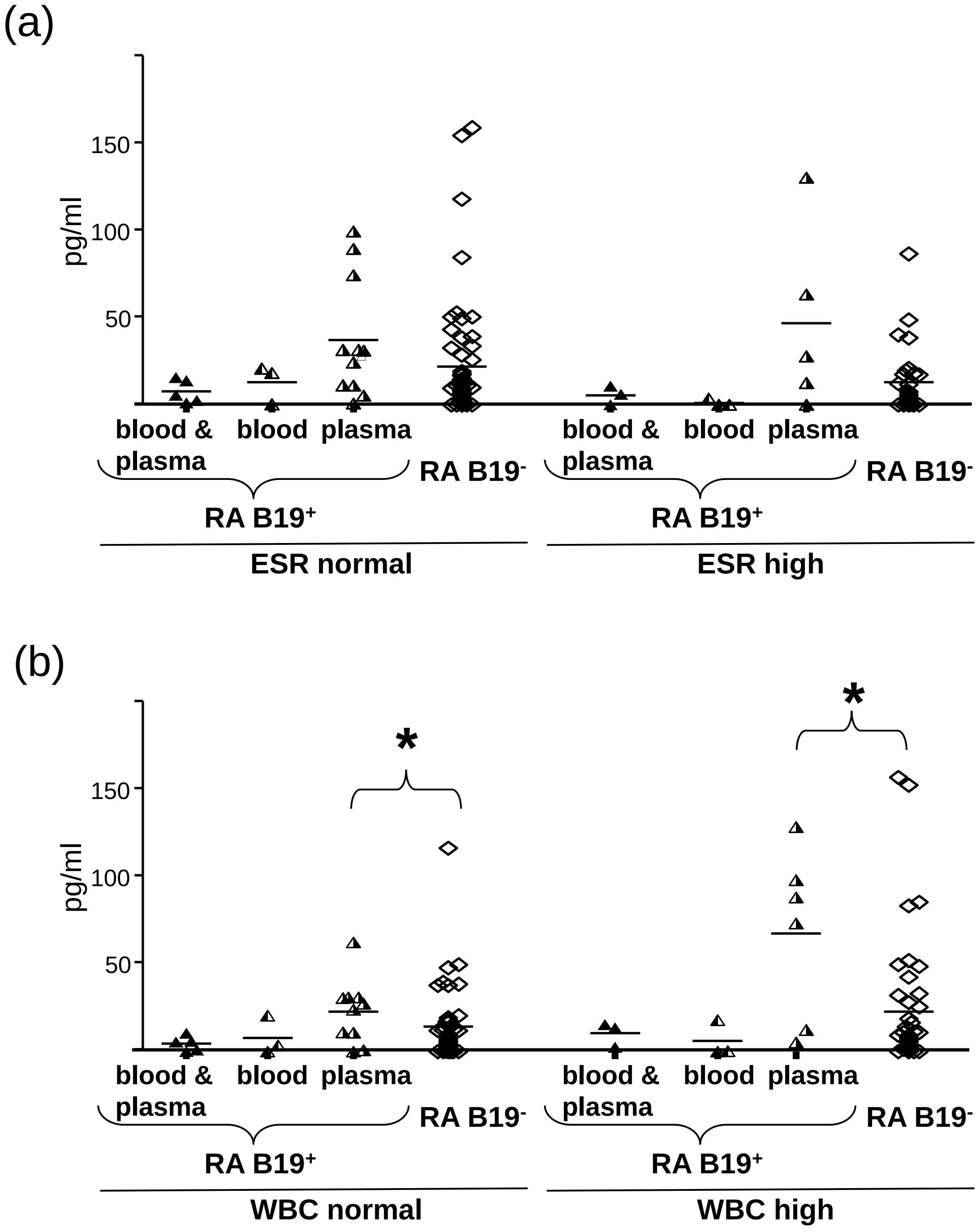

Fig. S2. The amount of IL-6 in the plasma of parvovirus B19 DNA positive ($B19^+$) and parvovirus B19 negative ($B19^-$) rheumatoid arthritis patients.
The patients are divided into the groups accordingly to the findings of virus DNA (in blood and cell-free plasma, blood or cell-free plasma) and normal and high levels of ESR high (a); normal and high levels of WBC (b); αCCP<60 and αCCP>60 (c); RF<250 and RF>250 (d). One symbol shows the amount of IL-6 (pg /mL) of one person. The results significant according to Mann-Whitney U test are marked by stars: p<0.05 (*) or p<0.005 (**)

Fig. S2

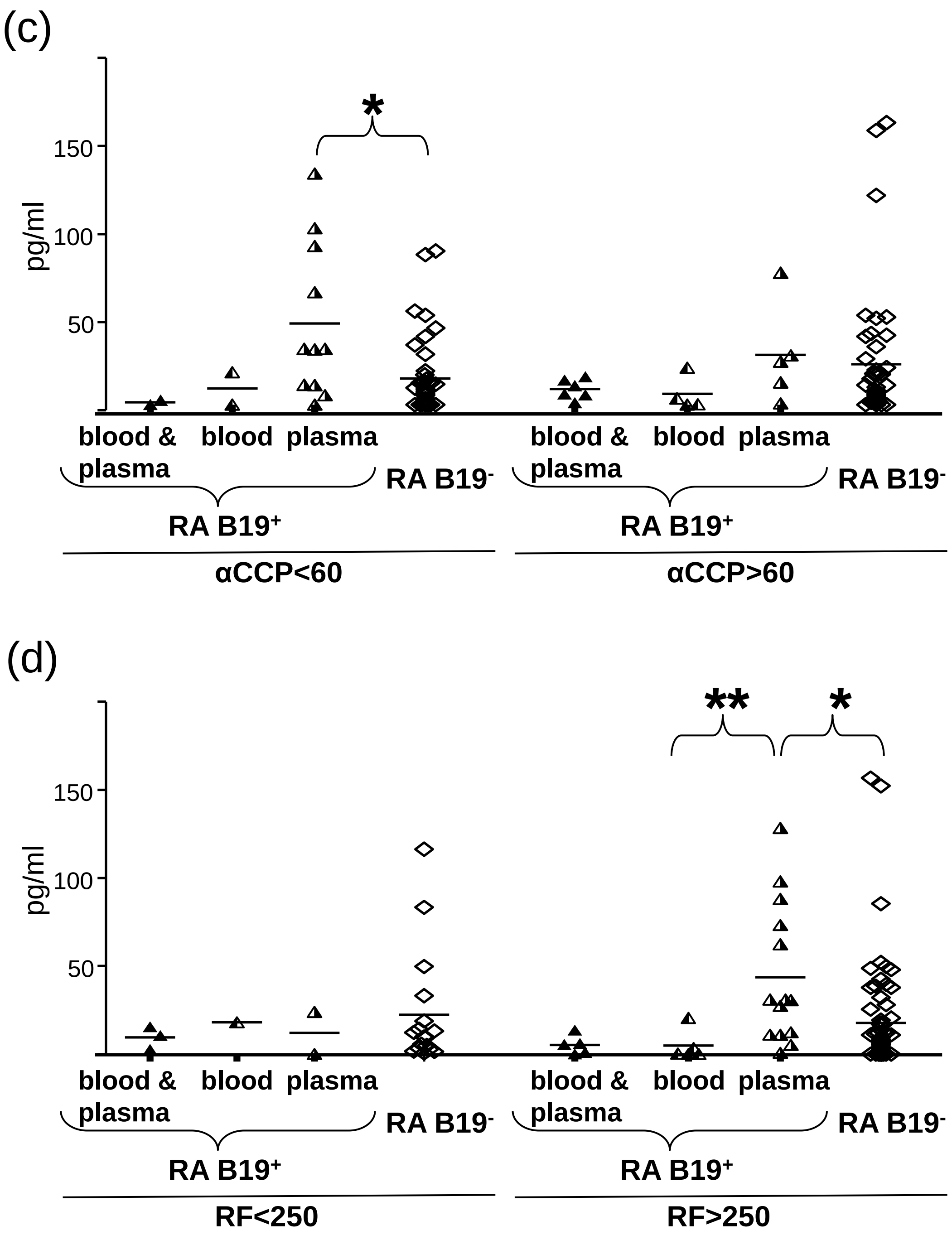

(c)
pg/ml
150
100
50
*
blood & plasma
blood
plasma
RA B19-
RA B19+
αCCP<60
αCCP>60
(d)
pg/ml
150
100
50
**
*
blood & plasma
blood
plasma
RA B19-
RA B19+
RF<250
RF>250

Table S1. Parvovirus B19 infection markers in rheumatoid arthritis patients and healthy persons

Parvovirus B19 infection marker/s	Numbers of		IL-6 concentration, pg/ml		Infection
	RA patients	Healthy blood donors	RA patients	Healthy blood donors	
Blood DNA positive & IgM negative & IgG positive	5	7	8.7±10.2	1.0±1.2	Persistent infection in latent phase
Blood DNA positive & IgM negative & IgG negative	1	1	0	0	Persistent infection in latent phase
Plasma DNA positive & IgM negative & IgG positive	15	0	41.3±41.1	-	Persistent infection in active phase
Plasma DNA positive & IgM negative & IgG negative	1	1	31.8	0	Could be acute infection
Blood DNA and plasma DNA positive & IgM negative & IgG positive	7	0	6.5±6.2	-	Persistent infection in active phase
Blood DNA and plasma DNA positive & IgM negative & IgG negative	1	0	10.8	-	Acute or persistent infection in active phase
DNA negative & IgM negative & IgG positive	76	32	20.4±32.2	2.0±3.8	Past infection
DNA negative & IgM negative & IgG negative	12	8	10.4±13.0	0.5±1.4	Without infection
**Number of analysed**	**118**	**49**			

Table S2. The amount of antibodies in sera of RA patients and control persons detected by Biotrin ELISA and Microgen RecomLine dot blot

Patients, code	B19V DNA detected		Biotrin ELISA		Microgen, RecomLine											
					IgM						IgG					
	blood	plasma	IgM	IgG	VP-2P	VP-N	VP-1S	VP2r	VP-C	NS-1	VP-2P	VP-N	VP-1S	VP2r	VP-C	NS-1
**RA. B19^{+}b/p**																
T95	+	+	0.4	**7.9**	**3.4**	**3.2**	0.6	0	0	0	**10.9**	**12.7**	**15.5**	**2.6**	0.7	0
T118	+	+	0.2	**7.9**	**1.9**	**3.6**	0.1	0	0	0	**7.6**	**13.6**	**15.1**	**2.1**	0	0.7
T105	+	+	0.2	**3.6**	0.7	0	0	0	0	0	**1.5**	0.4	0.1	0	0	0
T94	+	+	0.1	0.3	0	0.5	0	0	0	0	0	0	0	0	0	0
**RA. B19^{+}b**																
T123	+		0.2	**7.6**	**3.2**	0	0	0	0	0	**6.4**	**8.7**	**9.1**	0.4	0.2	0.1
T121	+		0.2	**7.4**	1.0	0.2	0	**1.1**	0	0.2	**3.9**	**1.5**	**1.3**	0	0	0
T133	+		0.1	**5.4**	**4.1**	1	0	0.6	0	0	**7.4**	**3.5**	**2.7**	**1.5**	0	0
T142	+		0.2	**2.4**	0	0.4	0	0	0	0	**1.5**	**8.5**	**7.9**	0	0	0
T109	+		0.2	**5.4**	0	0	0	0	0	0	**1.4**	0.3	0.0	0	0	0
**RA. B19^{+}p**																
T104		+	0.2	**8.8**	**1.1**	**5.8**	**3.1**	0	0	0	**10.6**	**20.6**	**26.5**	**1.7**	0	0
T122		+	0.1	**7.5**	ND	ND	ND	ND	ND	ND	**2.7**	**3.9**	**3.2**	**2.0**	0.9	0
T101		+	0.2	**7.3**	**1.2**	**3.5**	**1.2**	0.5	0.5	0	**4.7**	**9.7**	**11.8**	0	0	0
T128		+	0.1	**4.6**	0.5	0	0	0	0	0	**4.3**	**1.9**	**2.1**	0	1.0	0
T 99		+	0.2	**1.7**	ND	ND	ND	ND	ND	ND	0.9	**1.2**	**1.7**	0.9	0	0
T116		+	0.1	**5.2**	0	0	0	0	0	0	**2.8**	**1.1**	0.7	0	0	0
T97		+	0.1	**7.3**	0	0	0	0	0	0	**2.8**	0.7	0	0	0	0
T103		+	0.1	**4.6**	0	0	0	0	0	0	**2.0**	0	0	0	0	0
T106		+	0.1	**2.3**	0	0	0	0	0	0	**1.1**	0.7	0	0	0	0
T98		+	0.2	**1.7**	0	0	0	0	0	0	**1.2**	0.9	0.6	0	0	0
**T100**		+	0.3	**3.0**	ND	ND	ND	ND	ND	ND	0.9	0	0	0	0	0
T93		+	0.1	**1.8**	0	**2.5**	0.4	0.5	0	0	1.0	0	0	0	0	0
T102		+	0.1	0.1	ND	ND	ND	ND	ND	ND	0.6	0	0	0	0	0

	**RA. B19^{-}**														
T115		0.2	**8.4**	**1.1**	**2.2**	0	0	0	0	**6.7**	**9.5**	**10.8**	**4.1**	0	0
T108		0.1	**8.2**	**3.8**	0	0	0	0	0	**7.4**	**6.2**	**6.5**	**5.3**	**2.7**	0
T107		0.3	**7.8**	**2.5**	0.3	0	**1.7**	0	0	**5.7**	**3.7**	**3.5**	**1.7**	0.2	0
T117		0.1	**7.4**	0	0	0	0	0	0	**5.0**	**2.7**	**2.8**	**3.2**	0.9	0.6
T119		0.1	**6.2**	0.3	0	0	0	0	0	**5.0**	**6.4**	**6.4**	**1.6**	0.4	0
T124		0.4	**7.9**	0.4	0.2	0	0.3	0	0.3	**7.0**	**3.5**	**3.4**	0	0	0
T113		0.2	**6.8**	0	0.3	0	0	0	0	**4.2**	**2.9**	**1.8**	0	0	0
T138		0.2	**6.8**	**4.4**	0	0	0	0	0	**4.8**	**4.0**	**4.1**	0	0	0
T126		0.1	**6.8**	**4.6**	0.5	0	0	0	0	**7.0**	**8.6**	**9.9**	0.5	0	0
T110		0.1	**6.6**	0.7	**6.2**	0	0	0	0	**3.4**	**12.6**	**15.3**	0	0	0
T112		0.2	**6.3**	0.3	0.4	0	0	0	0	**4.1**	**2.7**	**2.6**	0	0	0
T 89		0.2	**4.4**	ND	ND	ND	ND	ND	ND	**1.3**	**2.9**	**2.8**	0	0	0
T134		0.1	**4.3**	ND	ND	ND	ND	ND	ND	**1.2**	**3.1**	**2.5**	0.6	0	0
T 81		0.2	**4.0**	ND	ND	ND	ND	ND	ND	**1.5**	**2.0**	**1.4**	0	0	0
T132		0.1	**5.1**	ND	ND	ND	ND	ND	ND	**1.7**	**1.3**	0.7	0.4	0.5	0
T 96		0.2	**4.9**	ND	ND	ND	ND	ND	ND	**2.7**	**1.3**	0.5	0	0	0
T 80		0.2	**1.9**	ND	ND	ND	ND	ND	ND	0.8	**1.2**	0.7	0	0	0
T 28		0.2	**1.2**	ND	ND	ND	ND	ND	ND	0.8	**1.1**	0	0	0	0
T114		0.1	**6.9**	0.4	0	0	0	0	0	**2.7**	0.6	0	0	0	0
T139		0.2	**5.5**	ND	ND	ND	ND	ND	ND	**1.4**	0.8	0.6	0	0	0
T111		0.1	**5.0**	ND	ND	ND	ND	ND	ND	**1.3**	0.6	0.6	0	0	0
T125		0.1	**4.9**	ND	ND	ND	ND	ND	ND	**1.2**	0.7	0.6	0	0	1.1
T131		0.1	**3.8**	ND	ND	ND	ND	ND	ND	**1.1**	0.5	0	0	0	0
T 31		0.2	**2.9**	ND	ND	ND	ND	ND	ND	**1.3**	0.9	0.7	0	0.7	0.5
T 43		0.2	**4.4**	ND	ND	ND	ND	ND	ND	1.0	0.8	0.7	0	0	0
T127		0.2	**5.6**	ND	ND	ND	ND	ND	ND	1.0	0	0	0.8	1.0	0
T135		0.2	**4.3**	ND	ND	ND	ND	ND	ND	1.0	0.6	0	0	0.7	0
T 84		0.2	**2.9**	ND	ND	ND	ND	ND	ND	0.9	0	0	0	0	0
T140		0.6	**2.5**	ND	ND	ND	ND	ND	ND	0.8	0.9	0.7	0	0	0
T 3		0.0	**2.5**	ND	ND	ND	ND	ND	ND	0.7	0	0	0	0	0
T130		0.2	**1.5**	ND	ND	ND	ND	ND	ND	0	0	0	0	0	0
T152		0.1	0.6	ND	ND	ND	ND	ND	ND	0	0	0	0	0	0
T120		0.1	0.4	ND	ND	ND	ND	ND	ND	0.8	0	0	0	0	0

**Control. B19^{+}**																
K45	+		0.1	**8.1**	0.5	0.4	0.4	0.3	0	0	4.9	**2.8**	**2.5**	0.7	0	0
K20	+		0.1	**7.8**	0	0	0	0	0	0	**5.2**	**3.3**	**3.1**	0.9	0.4	0.7
K9	+		0.1	**4.3**	0	0	0	0	0	0	**2.2**	**2.4**	**1.8**	0	0	0
K39	+		0.2	**3.0**	0.8	0.7	0.7	0.9	0	0	1.9	**3.6**	**3.4**	0.1	0	0.3
K41	+		0.2	**2.9**	0	0	0	0	0	0	1.2	**7.5**	**9.4**	0	0	0
K37	+		0.1	**3.3**	0	0	0	0.5	0	0	2.7	1.0	**1.8**	0	0	0
K24	+		0.1	**3.4**	0	0.5	0	0	0	0	**2.5**	**1.1**	0.6	0.4	0	0.3
K36	+		0.2	0.9	0.4	0.5	0	0.4	0	0	0	0.4	0	0	0	0
K22		+	0.1	0.4	0.3	0.3	0	0.4	0	0	0	0	0	0	0	0.6
**Control. B19^{-}**																
K15			0.4	**9.0**	0.4	0.9	0	0.4	0	0	**8.1**	**5.2**	**5.8**	**2.6**	1.0	**4.0**
K40			0.1	**8.6**	0.4	0	0	0	0	0	**6.8**	**5.4**	**5.4**	**1.2**	0.4	0.7
K16			0.2	**8.4**	0.3	0.3	0	0.2	0	0	**8.8**	**12.8**	**13.6**	**2.0**	0	0
K35			0.1	**8.4**	0.3	0.3	0.3	0.5	0	0	**10.1**	**11.7**	**10.1**	0	0	0
K30			0.2	**8.2**	0.47	0	0	0	0	0	**5.6**	**7.1**	**8.8**	0.4	0	0.0
K32			0.1	**8.1**	0	0	0	0	0	0	**4.5**	**3.0**	**2.7**	1.1	**1.1**	**1.1**
K3			0.1	**8.0**	0.6	0.6	0.8	0	0	0	**3.9**	**1.3**	**1.2**	0.3	0	0
K26			0.1	**7.6**	0	0.5	0	0	0	0	**5.7**	**6.9**	**8.2**	0.7	0	0.1
K12			0.2	**7.1**	0.3	0.2	0	0	0	0	**6.8**	**3.6**	**2.3**	1.0	0.1	0.1
K2			0.1	**6.2**	0.5	0.5	0.5	0	0	0	**4.9**	**4.1**	**3.5**	0	0	0
K23			0.3	**5.1**	ND	ND	ND	ND	ND	ND	**2.5**	**1.9**	**1.2**	0.6	0	0
K5			0.1	**7.3**	0	0	0	0	0	0	**5.2**	0.9	0.3	0	0	0.1
T 57			0.2	**4.5**	ND	ND	ND	ND	ND	ND	**2.8**	0.9	0.8	0	0	0
K43			0.1	**3.3**	ND	ND	ND	ND	ND	ND	0.9	0	0	0	0	0.6
K 8			0.1	**2.9**	ND	ND	ND	ND	ND	ND	1.0	0	0	0	0	0
K44			0.1	**2.8**	ND	ND	ND	ND	ND	ND	0.7	0.5	0.4	0	0	0
K14			0.1	**2.2**	ND	ND	ND	ND	ND	ND	0.7	0	0	0	0	0
K 6			0.1	**1.3**	ND	ND	ND	ND	ND	ND	0	0	0	0	0	0
K 7			0.1	**1.2**	ND	ND	ND	ND	ND	ND	0	0	0	0	0	0

The amount of antibodies is presented as index value. The samples are positive, when the index value ≥1.1
Biotrin ELISA: the index was calculated as the ratio of sample's optical density to cutoff's optical density.

Microgen RecomLine: the blots were scanned and the band density was quantified using ImageJ 1.49 software. The index was calculated as the ratio of sample’s band density to cut off's band density. The amount of antibodies is marked as “0”, when the bands in RecomLine dot blot were not visible.

Table S3. The cytokine levels (pg/ml) in plasma of rheumatoid arthritis patients

Cytokine	B19V DNA positive			B19V DNA negative
	blood & cell-free blood plasma	blood	cell-free blood plasma	
IFNγ	64.16 ± 56.88	102.59 ± 88.18	61.58 ± 87.35	85.04 ± 64.37
IL-2	186.03 ± 208.82	24.33 ± 33.28	50.35 ± 59.88	54.28 ± 102.23
IL-4	17.59 ± 12.94	13.65 ± 16.84	23.25 ± 14.28	10.79 ± 11.12
IL-10	26.62 ± 12.92	73.66 ± 107.35	25.49 ± 15.61	53.79 ± 85.64
IL-12	7.14 ± 4.52	5.66 ± 5.57	10.95 ± 11.03	7.55 ± 8.54
IL-17	12.09 ± 16.86	16.73 ± 5.05	10.26 ± 8.17	13.72 ± 11.67
TNFα	9.32 ± 12.92	40.83 ± 40.83	21.21 ± 21.62	21.58 ± 29.91

Table S4. Spearman's rank correlation coefficient (r) between the amount of cytokines or antibodies in the blood plasma and clinical data

Correlation groups	Rheumatoid arthritis patients					Healthy persons	
	$B19^+$	$B19^+$ plasma& blood	$B19^+$ blood	$B19^+$ plasma	$B19^-$	$B19^+$	$B19^-$
Number of analysed	**30**	**8**	**6**	**16**	**88**	**9**	**44**
IL-2 / IL-12	**0.5 ***	-	0.5 ns	**0.91 *****	-	-	-
IL-10 / IL-12	0.5 **	-	-	**0.71 ****	0.5 ***	0.5 ns	0.6 ***
IL-12 / disease duration	-	-	0.6 ns	**0.54 ***	-	ND	ND
IL-12 / DAS28	-	-	-	**0.53 ***	-	ND	ND
IL-10 / CRP	0.6 **	-	0.7 ns	**0.62 ***	-	ND	ND
IL-4 / HgB	-	-	-	**-0.50 ***	-	ND	ND
IL-6 / HgB	-0.5**	-	-0.6 ns	**-0.69 ****	-	ND	ND
IL-6 / CRP	0.6 **	-	0.5 ns	**0.70 ****	-	ND	ND
IFNγ/ CRP	-	-	0.8 ns	**0.58 ***	-	ND	ND
Number of analysed	**22**	**4**	**5**	**13**	**33**	**9**	**19**
VP-C / IL-6	-0.5 ns	0.7 ns	- 0.7 ns	**-0.63 ***	-	-	-
VP-N/ age	-	-	-	**-0.6 ***	-	-	-
VP-1S/ age	-	-	-	**-0.6 ***	-	-	-
VP-2r/ age	-	-	-	**-0.61 ***	-	-	-
VP-2r / RF	-	-	0.6 ns	**-0.59 ***	-	ND	ND
VP-2P/ WBC	-	-	0.9 ns	**-0.56 ***	-	ND	ND

Spearman's rank correlation coefficient (r) is shown only from moderate to very strong correlation (r > 0.5 and r < -0.5). Low or negligible correlation (r) between -0.45 and +0.45 is marked as “-“.
The significant results are marked by starts: p<0.05 (*), p<0.005 (**) or p<0.0005 (***), ns- not significant. ND – not done.
